# Fluoroscopy-guided celiac plexus block – Trans-Aortic approach

**DOI:** 10.1016/j.inpm.2025.100644

**Published:** 2025-10-10

**Authors:** Kelly Li, Ivy Liu, Robert Jason Yong, David Hao

**Affiliations:** a Harvard Medical School, Boston, MA, USA; b Department of Anesthesiology, Mass General Brigham, Harvard Medical School, Boston, MA, USA

## Abstract

Transaortic celiac plexus block is an image-guided procedure used to manage refractory abdominal pain, often related to malignancy or chronic conditions like pancreatitis. This educational video demonstrates the transaortic approach for celiac plexus block under fluoroscopic guidance. The video reviews indications, relevant anatomy, procedural steps, and potential complications. This content is intended to supplement formal instruction and enhance understanding of a targeted technique used in the management of abdominal pain.

## Video related to this article

The following is the video related to this article:

Multimedia component 1
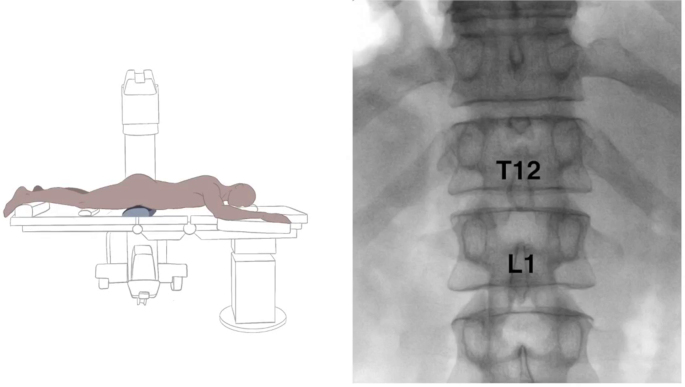

## Declaration of competing interest

The authors declare the following financial interests/personal relationships which may be considered as potential competing interests: Robert Jason Yong reports a relationship with Medtronic Inc that includes: consulting or advisory. If there are other authors, they declare that they have no known competing financial interests or personal relationships that could have appeared to influence the work reported in this paper.

